# Chromophore Quench-Labeling for Active Sites Counting in Ti-Based Ziegler–Natta Catalysts

**DOI:** 10.3390/polym17091211

**Published:** 2025-04-28

**Authors:** Antonio Vittoria, Giuseppe Antinucci, Roberta Cipullo, Vincenzo Busico

**Affiliations:** Department of Chemical Sciences, Federico II University of Naples, Via Cinthia, 80126 Napoli, Italy; giuseppe.antinucci@unina.it (G.A.); busico@unina.it (V.B.)

**Keywords:** chromophore quench labeling, catalytic olefin polymerization, Ziegler–Natta catalysts, active sites count

## Abstract

Chromophore quench-labeling (CQL) is an elegant and effective method to count the fraction of active metal (x*) in olefin polymerizations mediated by molecular transition metal catalysts. In this study, the method was successfully applied for the first time to a heterogeneous Ti-based Ziegler–Natta catalyst of industrial relevance. CQL experiments using 1-hexene as the monomer ended up with a value of x* = 0.49 ± 0.09%, close to that measured for the same catalyst in the polymerization of propene under otherwise identical conditions using an alternative quenched flow (QF) approach. We ascribe such a low x* value to the fact that the catalytically active species are transient metastable surface Ti adducts, as proposed in the recent literature.

## 1. Introduction

Elucidating the molecular kinetics of any chemical reaction—either stoichiometric or catalytic—is key for mechanistic understanding. Transition metal (M)-mediated olefin polymerizations are no exception in this regard [1,2]. The chain propagation step, namely olefin insertion into an M-C σ bond, can be written as:

M-P_y_ + C_n_H_2n_ → M-P_y+1_ (1)

(P_y_ = growing polymer chain with y monomeric units). The polymerization rate is usually expressed by Equation (2) [1]:

*R*_p_ = −d[C_n_H_2n_]/d*t* = 〈*k*_p_〉 x*[M][C_n_H_2n_] (2)

where 〈*k*_p_〉 is the apparent average kinetic constant of chain propagation, [M] is the analytical concentration of the metal, x* is the mole fraction of active metal, [C_n_H_2n_] is that of the monomer in the reaction phase, and *t* is the reaction time. Unfortunately, this seemingly simple expression has two major limitations when applied for mechanistic purposes. One is that *k*_p_ is a complex average over a population of active sites that widely differ in reactivity (even in the case of molecular catalysts, with the only possible exception of ethene homopolymerization, in which the active site is almost invariably a M-CH_2_ fragment), and as such, it is difficult to unravel [1,2,3,4,5]. The other is that, in a vast majority of cases, the value of x* is unknown, particularly with heterogeneous Ti-based Ziegler–Natta (Ti-ZN) catalysts, where the active sites are located on rather ill-defined surfaces [6,7,8,9].

**Figure 1:**
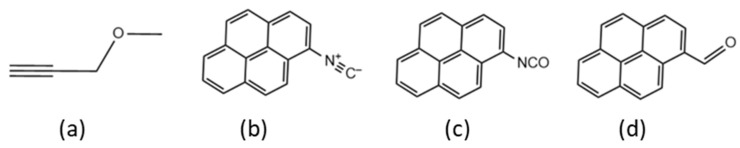
Possible QL agents (see text): (**a**) propargyl ether, (**b**) pyrene–isonitrile, (**c**) pyrene–isocyanate, (**d**) pyrene–aldehyde.

Two basic strategies to measure x* have been proposed in the literature. The ‘Quench-Labeling’ (QL) approach consists of the introduction and subsequent count of a label at the end of the growing polymer chains by means of a reaction that quenches chain propagation [7,10,11,12,13,14,15]. Typical labels are strong poisons, such as ^14^CO, ^13^CO, CH_3_O^3^H [16,17,18,19,20], (functional) alkynes (see e.g., Figure 1a) [7], or other unsaturated molecules bearing a UV chromophore or a fluorescent fragment (see e.g., Figure 1b–d) [10].

The use of radioactive QL agents, such as ^14^CO and CH_3_O^3^H, was popular until the 1970s [21], when spectroscopic detection was not adequately sensitive and safety concerns were less stringent. The main limitation of such labels is that the counting method is chemically ‘blind’, and one cannot rule out the hypothesis that the label-tagged molecules, other than growing polymeryls (e.g., ‘dead’ chains bonded to Al, formed by trans-alkylation between the catalyst and an alkyl-Al activator [21]), or multiple tagging of individual polymeryls occurred (e.g., alternated insertions of olefins and CO, later demonstrated by ^13^C NMR QL studies of *ansa*-zirconocene catalysts with ^13^CO [7,22]). Therefore, it is well possible that their use led to x* overestimations.

In recent years, the QL approach was revisited by introducing ‘smart’ labels easy/easier to evaluate spectroscopically and claimed to be unreactive with ‘dead’ Al-bound polymeryls. One such label is the propargyl ether (MPE) of Figure 1a. Group 4 M-R species are known to undergo single insertions of this alkyne with a Cossee-type mechanism [23,24], likely because the back-biting of the ether moiety shuts down metal reactivity. MPE has the advantage of being commercially available and has proved to be an efficient quench-labeling agent for growing poly(1-hexene) chains. It was first employed to active sites count of a representative single-center catalyst, e.g., Cp_2_ZrMe_2_, and then applied to study the kinetics of 1-hexene polymerization promoted by a prototype Ti-ZN catalyst. In both cases, the labeled chain ends were detected and quantified by means of ^1^H NMR spectroscopy, and the value of x* < 1 for the Ti-ZN system was calculated [7].

A number of ‘Chromophore Quench-Labels’ (CQL) (Figure 1b–d) have been proposed by Landis et al. [10,11]. The advantage of such labels is that a UV-Vis detector can be installed on a gel permeation chromatography (GPC) setup, in addition to a more conventional IR, RI, or LS detector, so that two molecular weight distribution (MWD) curves are obtained from one single CQL experiment: one corresponds to the overall MWD (i.e., includes labeled and unlabeled polymer chains); another refers exclusively to labeled chains. In case the two MWD curves coincide, one can rule out the hypothesis that the label reacted selectively with only part of the catalytic species. These CQL labels have only been applied to determine the x* value with single-center catalysts and have never been applied to heterogenous Ti-ZN systems.

The second strategy is based on ‘Quenched-Flow’ (QF) techniques. The idea, originally introduced by Keii and coworkers [25,26] (who referred to their method as ‘Stopped-Flow’) is to operate at the very early stages of the polymerization process, when chain growth is still within the controlled (‘pseudo-living’) kinetic regime (that is, before chain transfer processes become appreciable). Under this regime, the following equations, originally derived by Natta [17], hold:

(3) $$Y=R_{p}t=\langle k_{p}\rangle x^{*}\left[M\right]\left[C_{n}H_{2n}\right]t$$

(4) $$\frac{1}{P_{n}}=\frac{\langle f_{tr}\rangle }{\left(\langle k_{p}\rangle \left[C_{n}H_{2n}\right]\right)}+\frac{1}{\left(\langle k_{p}\rangle \left[C_{n}H_{2n}\right]\right)}\cdot \frac{1}{t}$$

where *R*_p_, *k*_p_, [M], [C_n_H_2n_], and x* are as defined in Equation (2), 〈*f*_tr_〉 is the average cumulative frequency of chain transfer events, and *P*_n_ is the number-average degree of polymerization. Measuring *P*_n_ = *f*(*t*) gives access to x*, 〈*k*_p_〉, and 〈*f*_tr_ 〉 by interpolating the experimental data points in terms of Equations (3) and (4). A technical drawback of the QF approach is that it requires operating at reaction times of the order of the average chain growth time, which for competent olefin polymerization catalysts is *t*_cg_ < 1 s, even under mild conditions.

QF measurements have been reported mainly for heterogeneous Ti-ZN catalysts, and invariably resulted in x* << 5% [25,26,27,28,29]. In particular, a recent study on propene polymerization at 40 °C in a heptane slurry in the presence of an industrially relevant Ti-ZN formulation with the composition MgCl_2_/Dibutylphthalate/TiCl_4_-Diisobutyldimethoxysilane/TEA (short notation **C1**; Ti = 2.0 wt%; TEA = AlEt_3_) yielded 〈*k*_p_〉 = (9.4 ± 0.9) × 10^3^ s^−1^ M^−1^, 〈*f*_tr_〉 = 6 ± 1 s^−1^, and x* = (0.21 ± 0.02)% [8]. Compared with the QL counts of x*~10–20% [20,21,30], that—as noted before—might represent overestimations. These QF counts can be suspected to be underestimations because they are made before catalyst particle fragmentation has occurred.

In view of the above, we considered it timely to run CQL experiments for heterogeneous Ti-ZN catalysts, which, to the best of our knowledge, were still unprecedented. In this paper, we report the results for the previously mentioned formulation **C1**.

## 2. Materials and Methods

All manipulations of air-sensitive compounds were conducted under argon or nitrogen using Schlenk techniques and/or MBraun LabMaster 130 glove boxes. Toluene and 1-hexene were dried by passing through a column packed with a commercially available Q-5 catalyst (13% Cu(II) oxide on Al_2_O_3_), stored inside the glove box over silica, and filtered before usage. The Ti-ZN precatalyst and diisobutyldimethoxysilane were kindly donated by SABIC and used as received. TEA was purchased from Chemtura and used as received. Pyrene–isonitrile (PyrNC) was synthesized according to the literature [31,32].

The 1-hexene polymerization protocol was as follows. In glass vials, solutions of TEA in toluene (250 μL, [Al] = 100 mM), kept at 40°C under magnetic stirring (500 rpm), were added with aliquots of solid precatalyst (1.25-5.0 mg, [Al]/[Ti] = 30). After 300 s, neat 1-hexene (125 μL, 84.1 mg) was added, reaching a total working volume of 1.0 mL (initial value of [1-hexene] = 1.0 M) and the polymerization started. After a desired time, the polymerization was quenched with the proper amount of PyrNC (50 mM solution in toluene, [PyrNC]/[Ti] = 1 to 24). The mixtures were left under stirring for at least 30 min. For the present study, a fixed precontact time between the catalyst and the Al cocatalyst was selected to prevent the extraction of the internal donor from the catalytic surface due to its reactivity with AlR_3_ [33,34].

The subsequent workup protocol was as follows. Each vial was added with 40 μL of a diphenylmethane solution in toluene (0.69 M) as a standard, and approximately 50 μL of the liquid phase were sampled out and analyzed by ^1^H NMR in CDCl_3_ to measure the amount of unreacted 1-hexene. Next, the reaction phase (including the aliquot used for ^1^H NMR analysis) was poured into an excess of MeOH to remove the unreacted PyrNC. The polymer was decanted, the supernatant was removed, and a second MeOH washing cycle was performed. The polymer was then recovered, dissolved in THF (1.0 mg mL^−1^), filtered using disposable syringe filters with a 0.2 μm pore size, and subjected to UV-GPC analysis.

Quantitative NMR spectra to measure 1-hexene monomer conversion were recorded at 298 K using a Bruker (Billerica, MA, USA) AV-400 MHz spectrometer fitted with a SmartProbe, using a relaxation delay of 10 s. Selected labeled polymers were characterized via ^13^C NMR with a Bruker (Billerica, MA, USA) Avance III 400 spectrometer equipped with a 5 mm high-temperature cryoprobe and a robotic sample changer with a pre-heated carousel (24 positions), following the acquisition protocols reported elsewhere [35,36].

The UV-GPC analyses were performed using a Viscotek (Worcestershire, UK) GPCmax/VE 2001 instrument fitted with PolyPore columns (2 × 300 × 7.5 mm) featuring a 5 µm particle size, from Polymer Laboratories. The polymer samples were eluted in THF solution at a flow rate of 1.0 mL/min at 40 °C and analyzed using a Viscotek (Worcestershire, UK) Model 302-050 Tetra Detector Array for differential refractive index (RI) and UV absorption (@ λ = 344 nm). Relative molecular weight calibrations were performed using a five-point narrow polystyrene (PS) standard (EasiCal PS-2 from Polymer Laboratories (Varian)). Omnisec software (v. 5.10, Viscotek, Worcestershire, UK) was used for initial data processing, which included positioning the baseline, setting peak integration limits, and applying the molecular weight calibration. UV data processing was carried out using Microsoft Excel, starting from the raw data in mV. All signals were processed by subtracting the dark spectrum and scaling by the background spectrum (typically performed using the built-in features of the spectrophotometer software, Omnisec v. 5.10). The chromophore concentration was calibrated for PyrNC using the standard Beer’s law. In all cases, the deviance of determination on the duplicate samples was always equal to or less than 20% (in line with the applied calibration method). Integration of the UV signal yields the concentration of labeled polymeryls, from which the fraction of active sites can be determined.

## 3. Results and Discussion

Catalyst **C1** is used industrially for the production of isotactic polypropylene (iPP). For the present study, however, we opted for 1-hexene as the monomer because the UV-GPC setup described in the previous section cannot operate with iPP at high temperature, while isotactic poly(1-hexene) (iPH) is amorphous at room temperature and soluble in THF at 40 °C.

Pyrene–isonitrile (PyrNC, Figure 1b) was used as the CQL agent because it can only coordinate to alkyl aluminum reagents without inserting into the Al−R (R = alkyl or hydrogen) bonds in polymerization conditions [12]. Therefore, selective labeling of Ti–polymeryls in the presence of Al reagents occurs with PyrNC.

**Figure 2:**
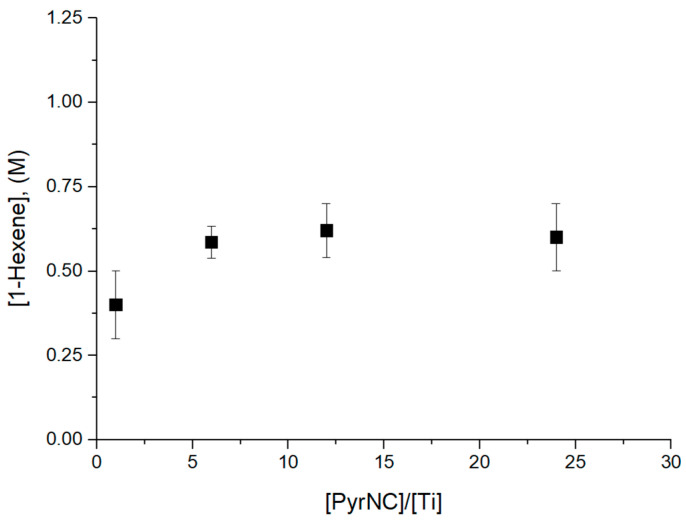
Monomer consumption as a function of [PyrNC]/[Ti] mole ratio for 1-hexene polymerization experiments at 40 °C, *t* = 90 s (see text).

Its quenching ability was tested in a series of preliminary 1-hexene polymerization experiments in the presence of catalyst **C1** at 40 °C and reaction time *t* = 90 s at a variable [PyrNC]/[Ti] molar ratio in the range of 1.0 to 24 (Table S1). We also performed quenching experiments at PyrNC/Ti > 24, but in this case, any attempt to completely remove the unreacted PyrNC failed. The UV signal of the labelled chains partially overlapped with the strong signal of the PyrNC, making the accurate x* determination not feasible. The reaction phases were characterized by ^1^H NMR spectroscopy to determine the concentration of unreacted 1-hexene (see Section 2). The results, reported in Figure 2, clearly indicated that the polymerization was effectively shut down at [PyrNC]/[Ti] ≥ 6.

**Figure 3:**
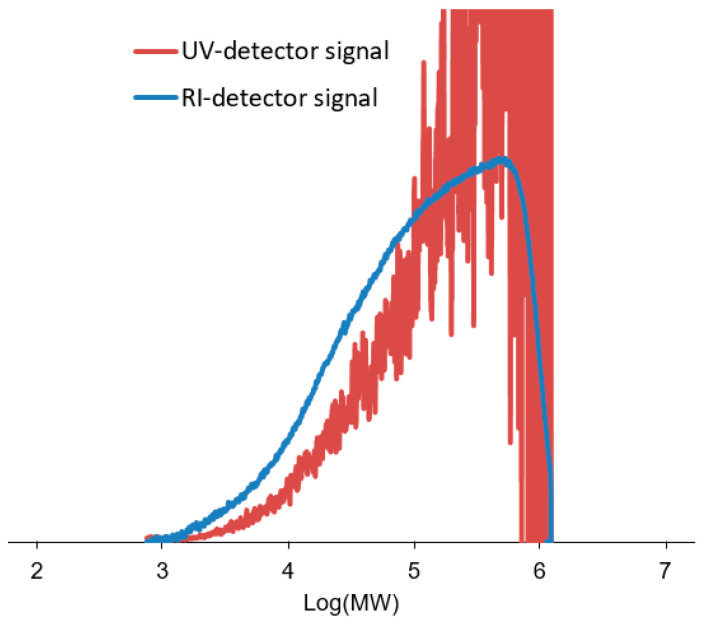
Overlay of MWD curves for a representative CQL poly(1-hexene) sample (#3 of Table 1) obtained by GPC with RI and UV-Vis detection (see text). For visualization purposes, the UV signal has been expanded vertically, and it is not on the same scale as the RI signal.

**Figure 4:**
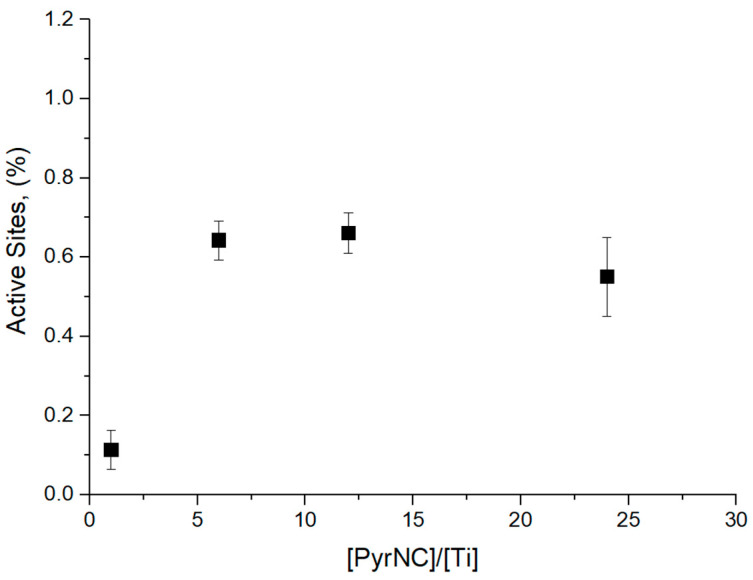
Values of x* measured by UV-GPC as a function of [PyrNC]/[Ti] for CQL 1-hexene polymerization experiments at 40 °C and 90 s reaction time (see text and Figure 2).

The polymer samples, coagulated with methanol and dried under vacuum (see Section 2), were characterized by GPC using both UV-Vis and RI detectors. As already noted in the Introduction, the RI detector measured the overall MWD, whereas the UV-Vis detector provided the MWD of the PyrNC-labeled chains only (Figure 3). The much lower signal-to-noise (S/N) ratio of the latter curve is a first qualitative indication of a low x* value. Consistently, quantitative integration of the UV-Vis signal yielded x* values in the range of 0.4–0.7%, independently of the [PyrNC]/[Ti] ratio (Figure 4). On the other hand, the overall MWD of the two curves turned out to be qualitatively similar, which indicates that quenching by PyrNC was almost unselective, notwithstanding the high polydispersity (*M*_w_/*M*_n_ > 5) typical of Ti-ZN polyolefin samples. This is an important indication that the CQL method can be used with good confidence, not only for active sites count with molecular catalysts but also with heterogeneous Ti-ZN ones. The fact that the two traces are basically superimposable clearly indicates that the method is effectively labeling all Ti species bearing a polymeryl chain (irrespective of the regiochemistry of the last inserted monomeric unit) at the moment of quench.

In line with the low x* value, PyrNC-labeled chain ends could not be detected by ^1^H and ^13^C NMR spectroscopy, even when using a spectrometer equipped with a high-temperature cryoprobe [36].

Preliminary experiments at variable [Al]/[Ti] ratios (from 10 to 60) were also carried out. The optimal productivity was obtained with Al/Ti ≥ 30, which is in line with what was previously found by others in Ti-ZN 1-hexene polymerization [7]. For the quenching experiments, working at [Al]/[Ti] = 30 or 60 did not affect the value of x*.

After optimizing the experimental quenching protocol, a series of CQL experiments of 1-hexene polymerization with catalyst **C1** (*m*_cat_ = 2.0 mg) at 40 °C, [Al]/[Ti] = 30 and [PyNC]/[Ti] = 6, were performed at variable reaction times (between 5 and 300 s), to highlight a possible time dependence of x*. The results, summarized in Table 1, indicated that this was not the case: indeed, the value of x* was practically constant (0.49 ± 0.09%) in the explored time range. Importantly, the CQL estimate of x* is nicely consistent with the QF one [8], thus confirming the tendency of the QL method to overestimate x* when the QL agent can react with ‘dead’ Al-bound polymer chains [21].

**Figure 5:**
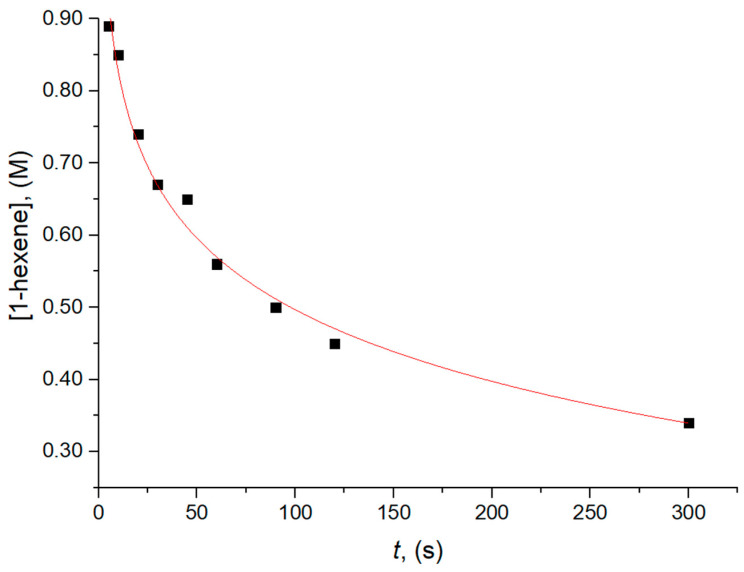
Monomer consumption vs. reaction time for CQL 1-hexene polymerization experiments at 40 °C (see text and Table 1).

The data on the polymer yield vs. polymerization time for the series of batch experiments in Table 1 could be well-reproduced by assuming a first-order dependence of the monomer insertion rate on monomer concentration (Figure 5), which agrees with the previous literature [17,37]. Decreasing polymer *M*_n_ with an increasing polymerization time was observed. This trend, since x* is the same in all experiments, can be ascribed to a lower chain propagation rate, due to some deactivation processes, or most likely to monomer depletion, as opposed to a monomer-independent chain transfer process (most likely, trans-alkylation with TEA).

From the experimental rate of polymerization at *t*→0 and the average value of x* = 0.49 ± 0.09% (Table 1), we calculated the average value of $\langle k_{p}\rangle$ = 3.4 × 10^3^ s^−1^ M^−1^. Such value is moderately lower than the corresponding one measured in QF propene polymerization experiments with the same catalyst formulation under very similar conditions ($\langle k_{p}\rangle$ = (9.4 ± 0.9) × 10^3^ s^−1^ M^−1^), which is very plausible in view of the different sizes of the two monomers.

## 4. Conclusions

In the present work, we have shown that the CQL method of active sites counting in catalytic olefin polymerization, originally implemented by Landis and coworkers for molecular catalysts [10,11,12,13,14,15], can be confidently extended to heterogeneous Ti-ZN ones.

For a typical Ti-ZN formulation, the value of x* in 1-hexene polymerization is very low (<1%), which is consistent with previous QF/NMR estimates in propene polymerization [7,8].

The agreement with previous independent QF estimates, in our opinion, is truly remarkable and suggests that the QF results are not flawed. It has long been suspected that the QF approach underestimates x* because of the very short experiment times (a few s) typical of the method, the catalyst particles cannot undergo fragmentation, and therefore, part of the Ti is not accessible to the monomer. This, however, cannot be the case for the present CQL study, with longer experimental times (up to 5 min) and polymer yields up to ca. 30 g g(catalyst)^−1^, at which some fragmentation does occur. Moreover, the precatalyst activation studies demonstrated that, without producing polymer, and therefore causing any fragmentation, the entire catalyst surface is accessible to TEA in less than 10 min [35].

Our interpretation is that x* measured by QF or QL corresponds to the fraction of Ti bearing a polymeryl (either growing or ‘dormant’ due to a last-inserted 2,1 monomeric unit) at any given moment. What escapes the count instead are the Ti species with Ti-R (e.g., R = Et) and Ti-H bonds, which are less sterically crowded and can form relatively stable adducts with AlR_3-x_Cl_x_ species and/or co-adsorbed donors [38,39].

**Figure 6:**
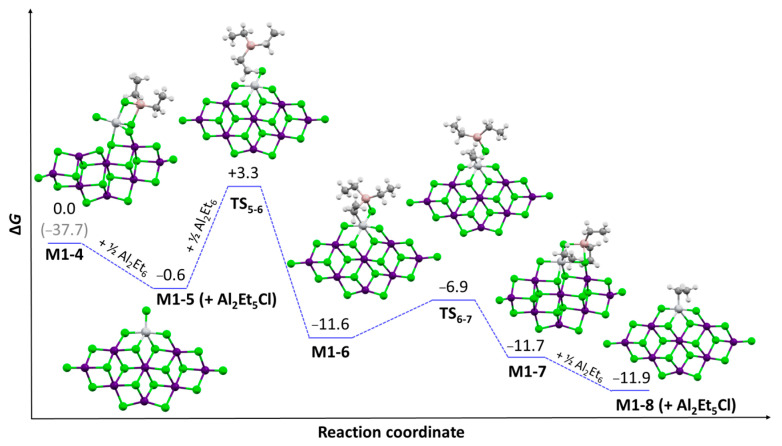
Models of adducts between adsorbed Ti(III) and AlR_3-x_Cl_x_ species. Figure adapted from Ref. [38]. Color key: Cl in green, Mg in violet, Ti in light gray, Al in pink, C in dark gray, and H in light gray (small spheres).

Examples of the former case can be found in Figure 6. All such adducts can ultimately change into active sites as soon as they open up, thus liberating the coordination site necessary for the π-coordination of the monomer. The process is highly endergonic, and the steady-state fraction of adducts *x*_add_ can be expected to be (much) larger than x*. Indeed, a high-resolution EPR investigation of catalyst **C1** after activation with AlMe_3_ vapor ended up with *x*_add_ ca. 10% [40].

In conclusion, the findings reported in the present work demonstrate that the approach is generally viable and could be extended to other types of olefin polymerizations as well, overcoming the limitations of using amorphous polyolefins by e.g., coupling the UV-Vis detector with more modern polymer characterization techniques [35,41,42,43].

## Figures and Tables

**Table 1:** Main results of the CQL 1-hexene polymerizations experiments at 40 °C and variable reaction time (see text). All results are averages of duplicate experiment pairs.

*Entry #*	*t* (s)	*Y* (mg)	*M*_n_ (kDa)	*M*_w_ (kDa)	PDI	x* (%)
1	5	9	84	480	5.7	0.40
2	10	13	73	411	5.6	0.68
3	20	22	81	469	5.8	0.36
4	30	28	68	419	6.2	0.42
5	45	29	70	390	5.6	0.48
6	60	37	67	384	5.7	0.52
7	90	42	49	341	7.0	0.55
8	120	46	47	346	7.4	0.52
9	300	56	38	265	7.0	0.52

## Data Availability

The original contributions presented in the study are included in the article and the Supplementary Materials; further inquiries can be directed to the corresponding author.

## Supplementary Materials

The following supporting information can be downloaded at https://www.mdpi.com/article/10.3390/polym17091211/s1: Table S1. Results of preliminary 1-hexene polymerization experiments at variable [PyrNC]/[Ti] molar ratio.

## References

[1] Kissin Y.V. (1985). Isospecific Polymerization of Olefins.

[2] Busico V., Kaminsky W. (2013). Giulio Natta and the Development of Stereoselective Propene Polymerization. Polyolefins: 50 Years After Ziegler and Natta I.

[3] Brintzinger H.H., Fischer D., Mülhaupt R., Rieger B., Waymouth R.M. (1995). Stereospecific Olefin Polymerization with Chiral Metallocene Catalysts. Angew. Chem. Int. Ed. Engl..

[4] Resconi L., Cavallo L., Fait A., Piemontesi F. (2000). Selectivity in Propene Polymerization with Metallocene Catalysts. Chem. Rev..

[5] Busico V., Cipullo R. (2001). Microstructure of Polypropylene. Prog. Polym. Sci..

[6] Cipullo R., Mellino S., Busico V. (2014). Identification and Count of the Active Sites in Olefin Polymerization Catalysis by Oxygen Quench. Macromol. Chem. Phys..

[7] Yu Y., Cipullo R., Boisson C. (2019). Alkynyl Ether Labeling: A Selective and Efficient Approach to Count Active Sites of Olefin Polymerization Catalysts. ACS Catal..

[8] Yu Y., Busico V., Budzelaar P.H.M., Vittoria A., Cipullo R. (2016). Of Poisons and Antidotes in Polypropylene Catalysis. Angew. Chem. Int. Ed..

[9] Chammingkwan P., Thang V.Q., Terano M., Taniike T. (2014). MgO/MgCl_2_/TiCl_4_ Core-Shell Catalyst for Establishing Structure-Performance Relationship in Ziegler-Natta Olefin Polymerization. Top. Catal..

[10] Nelsen D.L., Anding B.J., Sawicki J.L., Christianson M.D., Arriola D.J., Landis C.R. (2016). Chromophore Quench-Labeling: An Approach to Quantifying Catalyst Speciation As Demonstrated for (EBI)ZrMe_2_/B(C_6_F_5_)_3_-Catalyzed Polymerization of 1-Hexene. ACS Catal..

[11] Cueny E.S., Johnson H.C., Anding B.J., Landis C.R. (2017). Mechanistic Studies of Hafnium-Pyridyl Amido-Catalyzed 1-Octene Polymerization and Chain Transfer Using Quench-Labeling Methods. J. Am. Chem. Soc..

[12] Cueny E.S., Johnson H.C., Landis C.R. (2018). Selective Quench-Labeling of the Hafnium-Pyridyl Amido-Catalyzed Polymerization of 1-Octene in the Presence of Trialkyl-Aluminum Chain-Transfer Reagents. ACS Catal..

[13] Cueny E.S., Nieszala M.R., Froese R.D.J., Landis C.R. (2021). Nature of the Active Catalyst in the Hafnium-Pyridyl Amido-Catalyzed Alkene Polymerization. ACS Catal..

[14] Cueny E.S., Sita L.R., Landis C.R. (2020). Quantitative Validation of the Living Coordinative Chain-Transfer Polymerization of 1-Hexene Using Chromophore Quench Labeling. Macromolecules.

[15] Cueny E.S., Landis C.R. (2019). The Hafnium-Pyridyl Amido-Catalyzed Copolymerization of Ethene and 1-Octene: How Small Amounts of Ethene Impact Catalysis. ACS Catal..

[16] Natta G. (1959). Kinetic Studies of Alpha-Olefin Polymerization. J. Polym. Sci..

[17] Natta G., Pasquon I. (1959). The Kinetics of the Stereospecific Polymerization of α-Olefins. Adv. Catal..

[18] Shiono T., Ohgizawa M., Soga K. (1993). Reaction between Carbon Monoxide and a Ti-Polyethylene Bond with a MgCl_2_-Supported TiCl_4_ Catalyst System. Die Makromol. Chem..

[19] Feldman C.F., Perry E. (1960). Active Centers in the Polymerization of Ethylene Using Titanium Tetrachloride–Alkylaluminum Catalysts. J. Polym. Sci..

[20] Yaluma A.K., Tait P.J.T., Chadwick J.C. (2006). Active Center Determinations on MgCl_2_-Supported Fourth- and Fifth-Generation Ziegler–Natta Catalysts for Propylene Polymerization. J. Polym. Sci. Part A Polym. Chem..

[21] Mejzlík J., Lesná M., Kratochvíla J. (1986). Determination of the Number of Active Centers in Ziegler-Natta Polymerizations of Olefins. Catalytical and Radical Polymerization.

[22] Budzelaar P.H.M. (2004). CO/Ethene Copolymerization at Zirconocene Centers?. Organometallics.

[23] Eisch J.J., Piotrowski A.M., Brownstein S.K., Gabe E.J., Lee F.L. (1985). Organometallic Compounds of Group III. Part 41. Direct Observation of the Initial Insertion of an Unsaturated Hydrocarbon into the Titanium-Carbon Bond of the Soluble Ziegler Polymerization Catalyst Cp_2_TiCl_2_-MeAlCl_2_. J. Am. Chem. Soc..

[24] Clarke T.C., Yannoni C.S., Katz T.J. (1983). Mechanism of Ziegler-Natta Polymerization of Acetylene: A Nutation NMR Study. J. Am. Chem. Soc..

[25] Keii T., Terano M., Kimura K., Ishii K. (1987). A Kinetic Argument for a Quasi-Living Polymerization of Propene with a Magnesium Chloride-Supported Catalyst. Makromol. Chem. Rapid Commun..

[26] Taniike T., Sano S., Ikeya M., Thang V.Q., Terano M. (2012). Development of a Large-Scale Stopped-Flow System for Heterogeneous Olefin Polymerization Kinetics. Macromol. React. Eng..

[27] Terano M., Kataoka T. (1989). A Kinetic Study of Propene Polymerization Using MgCl_2_-supported Catalysts. Die Makromol. Chem. Rapid Commun..

[28] Mori H., Iguchi H., Hasebe K., Terano M. (1997). Kinetic Study of Isospecific Active Sites Formed by Various Alkylaluminiums on MgCl_2_-Supported Ziegler Catalyst at the Initial Stage of Propene Polymerization. Macromol. Chem. Phys..

[29] Matsuoka H., Liu B., Nakatani H., Terano M. (2001). Variation in the Isospecific Active Sites of Internal Donor-Free MgCl_2_-Supported Ziegler Catalysts: Effect of External Electron Donors. Macromol. Rapid Commun..

[30] Yang H., Zhang L., Zang D., Fu Z., Fan Z. (2015). Effects of Alkylaluminum as Cocatalyst on the Active Center Distribution of 1-Hexene Polymerization with MgCl_2_-Supported Ziegler–Natta Catalysts. Catal. Commun..

[31] Wang X., Wang Q.G., Luo Q.L. (2015). Synthesis of Isonitriles from N-Substituted Formamides Using Triphenylphosphine and Iodine. Synthesis.

[32] Wanzlick H.W., Lehmann-Horchler M., Mohrmann S., Gritzky R., Heidepriem H., Pankow B. (1964). New Methods of Preparative Organic Chemistry IV. Angew. Chem. Int. Ed. Engl..

[33] Zaccaria F., Vittoria A., Correa A., Ehm C., Budzelaar P.H.M., Busico V., Cipullo R. (2018). Internal Donors in Ziegler-Natta Systems: Is Reduction by AlR_3_ a Requirement for Donor Clean-Up?. ChemCatChem.

[34] Vittoria A., Antinucci G., Zaccaria F., Cipullo R., Busico V. (2020). Monitoring the Kinetics of Internal Donor Clean-up from Ziegler–Natta Catalytic Surfaces: An Integrated Experimental and Computational Study. J. Phys. Chem. C.

[35] Vittoria A., Meppelder A., Friederichs N., Busico V., Cipullo R. (2017). Demystifying Ziegler–Natta Catalysts: The Origin of Stereoselectivity. ACS Catal..

[36] Antinucci G., Vittoria A., Cipullo R., Busico V. (2020). Regioirregular Monomeric Units in Ziegler–Natta Polypropylene: A Sensitive Probe of the Catalytic Sites. Macromolecules.

[37] Ystenes M. (1991). The Trigger Mechanism for Polymerization of α-Olefins with Ziegler-Natta Catalysts: A New Model Based on Interaction of Two Monomers at the Transition State and Monomer Activation of the Catalytic Centers. J. Catal..

[38] Antinucci G., Cannavacciuolo F.D., Ehm C., Budzelaar P.H.M., Cipullo R., Busico V. (2024). MgCl_2_-Supported Ziegler-Natta Catalysts for Propene Polymerization: Before Activation. Macromolecules.

[39] Antinucci G., Cipullo R., Busico V. (2023). Imagine Polypropylene. Nat. Catal..

[40] Morra E., Giamello E., Van Doorslaer S., Antinucci G., D’Amore M., Busico V., Chiesa M. (2015). Probing the Coordinative Unsaturation and Local Environment of Ti^3+^ Sites in an Activated High-Yield Ziegler–Natta Catalyst. Angew. Chem. Int. Ed..

[41] Monrabal B., Romero L., Mayo N., Sancho-Tello J. (2009). Advances in Crystallization Elution Fractionation. Macromol. Symp..

[42] Antinucci G., Pucciarelli A., Vittoria A., Zaccaria F., Urciuoli G., Ehm C., Cannavacciuolo F.D., Cipullo R., Busico V. (2023). Fast Analytics of High-Impact Polypropylene (HIPP). ACS Appl. Polym. Mater..

[43] Vittoria A., Urciuoli G., Costanzo S., Tammaro D., Cannavacciuolo F.D., Pasquino R., Cipullo R., Auriemma F., Grizzuti N., Maffettone P.L. (2022). Extending the High-Throughput Experimentation (HTE) Approach to Catalytic Olefin Polymerizations: From Catalysts to Materials. Macromolecules.

