# Supplementary material for: Chromophore Quench-Labeling for Active Sites Counting in Ti-Based Ziegler–Natta Catalysts
Source: Polymers (Basel). 2025 Apr 28;17(9):1211. doi: 10.3390/polym17091211 (PMC12073164; doi:10.3390/polym17091211)
Supplement: Supplementary file 1 [file polymers-17-01211-s001.zip › polymers-3572468-supplementary.pdf]

Supporting Information

Chromophore quench-labeling for active sites counting in Ti-based Ziegler-Natta catalysts

Antonio Vittoria[*], *Giuseppe Antinucci, Roberta Cipullo*[*] *and Vincenzo Busico*

Department of Chemical Sciences, Federico II University of Naples, via Cinthia, 80126 Napoli, Italy

[*]Correspondence: antonio.vittoria@unina.it (A.V.); rcipullo@unina.it (R.C.)

Table S1. Results of preliminary 1-hexene polymerization experiments at variable [PyrNC]/[Ti] molar ratio.

[PyrNC]/[Ti]	$[\text{1-hexene}]_{\text{residual}}$, (M)
1	0.42
1	0.38
6	0.56
6	0.61
12	0.62
12	0.62
24	0.59
24	0.61

Experimental conditions: T = 40°C; m_{cat} = 2.0 mg; [Al]/[Ti] = 30; $[\text{1-hexene}]_0$ =1.0 M; t_p = 90 s.
